# Evaluation of the surface properties of 4-(Decyloxy) benzoic acid liquid crystal and its use in structural isomer separation

**DOI:** 10.3906/kim-2101-13

**Published:** 2021-06-30

**Authors:** Birol IŞIK, Fatih ÇAKAR, Hüsnü CANKURTARAN, Özlem CANKURTARAN

**Affiliations:** 1 Department of Chemistry, Faculty of Arts and Science, Yıldız Technical University, İstanbul Turkey

**Keywords:** Inverse gas chromatography, liquid crystal, selectivity, surface properties

## Abstract

The selectivity of 4-(Decyloxy) benzoic acid (DBA) liquid crystal in surface adsorption region (303.2–328.2 K) and thermodynamic region (423.2 – 433.2 K) was investigated by inverse gas chromatography at infinite dilution (IGC-ID). The selectivity parameters of the structural isomer series named butyl acetate, butyl alcohol, and amyl alcohol series were calculated for the DBA using IGC-ID technique. Additionally, the surface properties including dispersive surface energy (gS D), free energy (DGA S), enthalpy (DHA S), and acidity-basicity constants were calculated with net retention volumes obtained from IGC-ID experiment results. When the DHA S and DGA S are constants, DBA surface was found to be an acidic character (KD/KA @ 0.89).

## 1. Introduction

The liquid crystal (LC) state, which is different from the known forms, was discovered in the 1888s during the studies of Reinitzer [1] and Lehmann [2] on some cholesterol esters. LC state, also called as the meso-phase, is known as a physical state located between the crystalline solid and the isotropic liquid phase. Although it is considered as a separate phase between the solid and liquid phase, it can have at least one property of the solid and liquid phase. In the LC structure, the spontaneous orientation of the molecules in a certain direction provides a geometric selectivity to the stationary phase [3–6]. The orientation of the molecules in the LC structure can also differentiate the properties and usage fields of the LC. LCs are widely used in sensor technology, technological devices, such as televisions, computers, tablets, and biological fields [7–10].

In the conventional gas chromatography (GC), certain stationary phases suitable for the chromatographic column studied are used. The probes in volatile form to be analysed in the GC are separated from each other depending on their polarity on conventional stationary phases. It is not possible to analyse high molecular weight and nonvolatile materials, such as LCs, polymers, composites, etc. in conventional GC [11–15]. Therefore, IGC-ID is a simple, low cost, high efficiency, and high accuracy technique developed to analyse such substances. This technique is based on filling the substances to be analysed into the chromatographic column as a stationary phase and retaining the probes passed over them in vapor form at different times [16–18]. 

Separation of the isomer series is crucial industrially. When LCs are used as stationary phase for separation of isomer series, generally better, more efficient results can be obtained compared to conventional stationary phases. By using the IGC-ID technique, faster and more accurate results can be obtained compared to conventional separation methods [19–23]. 

Surface properties are closely related to important physicochemical phenomena such as colloidal stability, stickiness, and wettability. Besides, the surface properties, especially the surface energy, is an extremely important parameters in understanding the interaction between the surface of the material and various probes [24–26]. Surface energy arises from unbalanced molecular forces on the surface of the materials. The surface energy of the materials can be analysed using liquid adsorption, flow microcalorimetry, and contact angle measurements. Since the application of these techniques is difficult and limited, IGC-ID has become a preferred technique by researchers [27–31]. 

In the scope of this study, DBA’s ability to separate isomer series including butyl acetate series (n-butyl acetate (nBAc), iso-butyl acetate (iBAc) and tert-butyl acetate (tBAc)), butyl alcohol series (n-butyl alcohol (nBAl), iso-butyl alcohol (iBAl) and tert-butyl alcohol (tBAl)) and amyl alcohol series (n-amyl alcohol (nAAl), iso-amyl alcohol (iAAl) and tert-amyl alcohol (tAAl)), and surface properties were investigated by IGC-ID technique. The selectivity of DBA was investigated in surface adsorption (303.2–328.2 K) and thermodynamic region (423.2–433.2 K). Additionally, the IGC-ID experiments were carried out to investigate the surface properties of DBA in relation to polar and nonpolar probes in surface adsorption region (303.2–328.2 K). Using the retention data obtained from IGC-ID experiments, the parameters used to determine the selectivity parameters and the surface properties were calculated. 

## 2. Theory of inverse gas chromatography at infinite dilution (IGC-ID)

### 2.1. The selectivity coefficient

To determine selectivity of materials, the net retention volumes ( *V* *_N_* ) in surface adsorption region and thermodynamic region should be calculated as main data. For volatile polar and nonpolar solvents used in the analysis, the *V* *_N_* is closely related to the interaction of these solvents with the materials [32–36]. *V* *_N_* is calculated as follows: 

*V* *_N_* *= Q.J.(t* *_R_* *– t* *_A_* *).T/T* *_f_* ** (1)

Here, *t* *_R_* and *t* *_A_* are retention times of volatile probes and air, respectively; *Q* is the volumetric flow rate; *T* and *T* *_f_* are the column and ambient temperature, respectively; *J* is James-Martin pressure correction factor.

The selectivity of the stationary phase contained in the chromatographic column can be calculated from the proportioning of the numerical difference between the retention times obtained from the IGC-ID experiments. Besides, selectivity coefficient can also be calculated from the ratio of *V* *_N_* calculated according to Eq. (1). The selectivity of stationary phase is determined depending on the size of the selectivity coefficient ( *a* ). This value is calculated as follows [37,38]:

a *= (t* *_R1_* *– t* *_A_* *) / (t* *_R2_* *– t* *_A_* *) = V* *_N1_* */ V* *_N2_* (2)

Here, *t* *_R1_* and *t* *_R2_* are the retention time of the first and second isomer from the isomer pairs, respectively; *t* *_A_* is the retention time of air; *V* *_N1_* and *V* *_N2_* are the net retention volume of the first and second isomer, respectively. 

### 2.2. Surface properties

In recent years, IGC-ID is commonly used for examining the surface properties of the materials. The standard free energy (D *G* *_A_* *º* ) value for the adsorption of volatile probes on the stationary phase is calculated with the help of the *V* *_N_* resulting from the interaction between probe and stationary phase [39–41]. D *G* *_A_* *º* is calculated as follows: 

D *G* *_A_* *º = – RT ln (V* *_N_* *) + K* (3) 

Surface energy is an extremely important parameter in explaining the interaction between stationary phase and volatile probes. The greater the surface energy, the more interactions between molecules. On the contrary, when this energy is low, the interaction decreases. Surface energy of the stationary phase (g *_S_* ) can be calculated as a sum of dispersive energy (g *_S_* *^D^* ) generated by weak interactions on surface and specific energy (g *_S_* *^S^* ) generated by strong interactions on surface [42–44]: 

g *_S_* *= g* *_S_* *^S^* *+ g* *_S_* *^D^* ** (4)

g *_S_* *^D^* of stationary phase is determined when non-polar probes are injected at Henry’s law region. This energy is due to dispersive interactions between molecules on the surface of the material and non-polar probe molecules [45]. g *_S_* *^D^* values can be calculated in the surface adsorption region according to the method proposed by Dorris–Gray [46] as follows:

g *_S_* *^D^* *= (* D *G* *_[CH2]_* *)* *^2^* */ 4(N* *_A_* *)* *^2^* *(a* *_[CH2]_* *)* *^2^* g *_[CH2]_* ** (5)

Here, *g* *_S_* *^D^* is the dispersive energy of the surface (mj/m^2^), D *G* *_[CH2]_* is the adsorption free energy of a methylene group, which is determined the slope of the plot between the number of alkanes versus *RTlnV* *_N_* values, *N* *_A_* is the Avogadro’s number, *a* *_[CH2]_* is the molecular area of a methylene group (0.06 nm^2^) and g *_[CH2]_* is the surface energy of a methylene group. g *_[CH2]_* values are calculated at any temperature (t ^o^C) as follows [47]:

g *_[CH2]_* *= 35.6 – 0.058t* (6)

Additionally, the method proposed by Schultz is widely used to calculate dispersive energy of surface [48]. This energy is calculated as follows:

– *RT ln (V* *_N_* *) = 2N* *_A_* *a(* g *_S_* *^D^* *)* *^0.5^* *(* g *_L_* *^D^* *)* *^0.5^* *+ K* (7)

Here, *a* is the cross-sectional area of the probes, *N* *_A_* is the Avogadro’s number, g *_L_* *^D^* is the dispersive energy of the probes. The *a* and g *_L_* *^D^* values were taken from the literature, and were listed in Table 1. g *_S_* *^D^* values of stationary phase can be calculated from the slope of plot between *RTlnV* *_N_* versus *a(* g *_L_* *^D^* *)* *^0.5^* of non-polar probes.

**Table 1:** The values of a and gLD for non-polar and polar probes.

Probes	a(x10–10 m2)	gLD (mj/m2)
n-Hexane (Hx)	51.5	18.4
n-Heptane (Hp)	57.0	20.3
n-Octane (O)	62.8	21.3
n-Nonane (N)	69.0	22.7
n-Decan (D)	75.0	23.4
Dichloromethane (DCM)	31.5	27.6
Chloroform (TCM)	44.0	25.9
Tetrahydrofuran (THF)	45.0	22.5
Ethyl acetate (EA)	48.0	19.6
Acetone (Ace)	42.5	16.5

D *G* *_[CH2]_* values are calculated as follows [49]:

D *G* *_[CH2]_* *= – RT ln (V* *_N,n_* */V* *_N,n+1_* *)* (8)

Here, *R* is the universal gas constant; *V* *_N,n_* and *V* *_N,n+1_* are the net retention volumes of two n-alkanes having *n* and *n+1* carbon atoms, respectively.

D *G* *_A_* *^S^* for the polar probes are calculated as follows:

D *G* *_A_* *^S^* *= – RT ln (V* *_N_* */V* *_N(ref)_* *)* (9)

When the studies are carried out at different temperatures, D *H* *_A_* *^S^* and D *S* *_A_* *^S^* values can be calculated as follows [50]:

 D *G* *_A_* *^S^* *=* D *H* *_A_* *^S^* *– T* D *S* *_A_* *^S^* (10)

The value of *DH* *_A_* *^S^* is linked with *K* *_A_* (donor or acidity group) and *K* *_D_* (acceptor or basicity group) parameters. This situation is due to the interactions that occur between probes and surfaces that do not have dispersive and entropic interactions. These values are calculated as follows [51,52]:

*–* D *H* *_A_* *^S^* *= K* *_A_* *(* D *N) + K* *_D_* (AN*) (11)

Here, *DN* is an electron donor or acidity number and AN* is an electron acceptor or basicity number determined by Gutmann [53]. By calculating the value of D *H* *_A_* *^S^* for polar probes, a linear plot is drawn between *–* D *H* *_A_* *^S^* */AN* ^*^ and D N/AN* . The values of *K* *_A_* and *K* *_D_* of solid materials can be obtained from the slope and intercept of the line, respectively. If *K* *_D_* */K* *_A_* *> 1* , the surface is considered to be a basic; whereas, if *K* *_D_* */K* *_A_* *< 1* , the surface is considered to be an acidic.

## 3. Materials and methods

All the properties of the chemicals used in this study are given in Table 2.

**Table 2:** Source, assay, and CAS registry numbers of the chemicals.

Chemicals	Source	CAS No	Assay
DBA	Sigma Aldrich	5519-23-3	0.980
nBAc	Supelco	123-86-4	≥ 0.995
iBAc	Sigma Aldrich	110-19-0	≥ 0.980
tBAc	Sigma Aldrich	540-88-5	≥ 0.990
nBAl	Sigma Aldrich	71-36-3	≥ 0.994
iBAl	Supelco	78-83-1	≥ 0.990
tBAl	Sigma Aldrich	75-65-0	≥ 0.990
nAAl	Sigma Aldrich	71-41-0	≥ 0.990
iAAl	Sigma Aldrich	123-51-3	≥ 0.990
tAAl	Supelco	75-85-4	≥ 0.990
Hx	Supelco	110-54-3	≥ 0.997
Hp	Supelco	142-82-5	≥ 0.990
O	Sigma Aldrich	111-65-9	≥ 0.990
N	Sigma Aldrich	111-84-2	≥ 0.990
D	Sigma-Aldrich	124-18-5	≥ 0.940
EA	Supelco	141-78-6	≥ 0.998
Ace	Supelco	67-64-1	≥ 0.998
DCM	Supelco	75-09-2	≥ 0.998
THF	Supelco	109-99-9	≥ 0.998
TCM	Supelco	67-66-3	≥ 0.998

All measurements in IGC-ID studies were carried out using an Agilent Technologies HP-6890N device combined with thermal conductivity detector (TCD) (Hewlett-Packard, Palo Alto, CA, USA). The stainless-steel column (1/8” o.d., 2.10 mm i.d. 10 m) was purchased from Alltech Associates, Inc. (Chicago, IL, USA). Chromosorb W (AW-DMCS-treated, 80/100 mesh) was used as the support material and obtained from Sigma Aldrich. The DBA liquid crystal was dissolved in the Chloroform, and Chromosorb W was added slowly. A homogeneous mixture was obtained by continuous stirring in heating controlling water bath, and the LC was coated on support. Silane-treated glass wool used to plug the ends of the column was obtained from Alltech Associates Inc (Deerfield, IL, USA). The ends of the column were loosely plugged with silanized glass wool. After the column was cut to a size of 1 m and cleaned thoroughly, approximately 1.21 g of the prepared column interior material was filled. The total loading of DBA liquid crystal on the support was determined as 10.38% by weighing. Helium (He), which kept at a constant flow rate of 3.6 mL/min, was used as the mobile phase during the experiments. Probes and air were injected into the column with 1 mL and 10 mL Hamilton syringes, respectively. For infinite dilution, the probe (0.1 mL) was taken into the syringe and flushed into the air. Then, the retention times for probe and air were determined. At least four consecutive injections were made for each probe and air at each set of measurements.

## 4. Results and discussion

**Figure 1:**
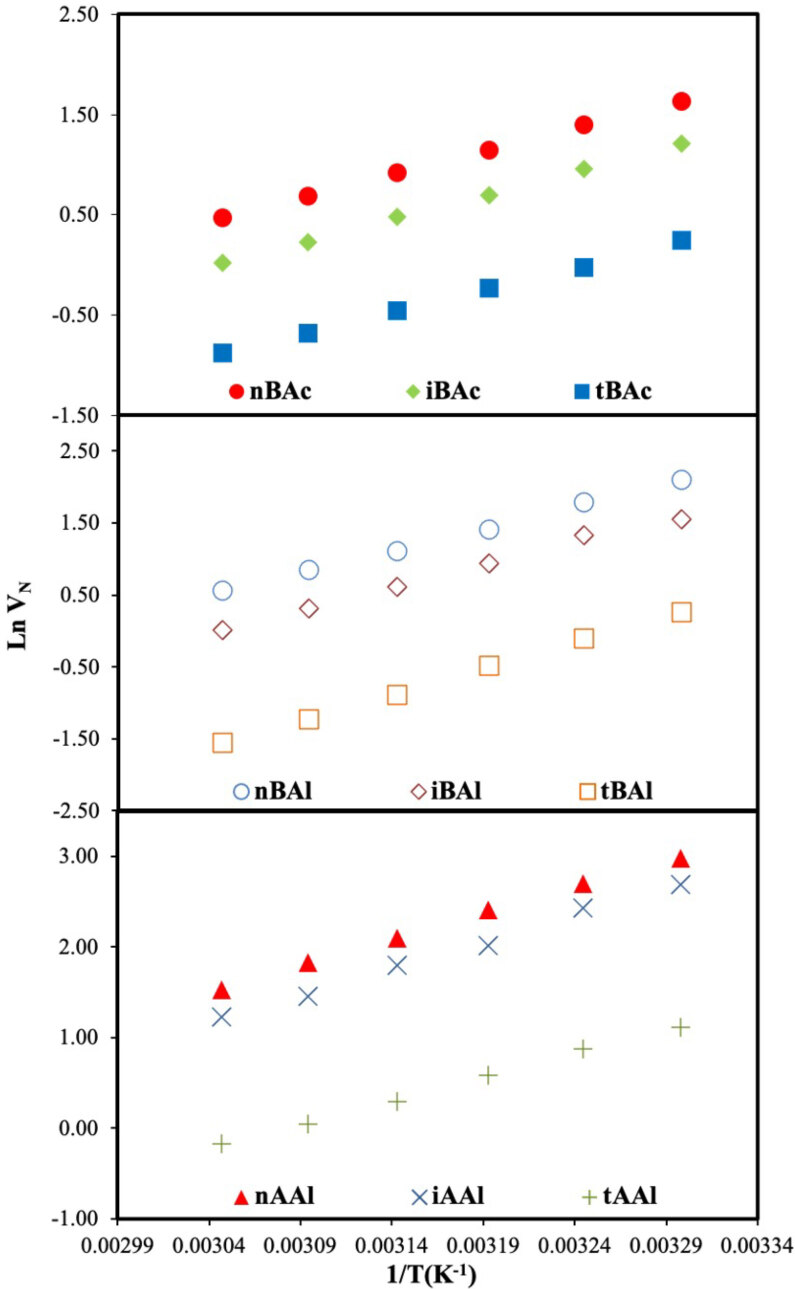
Net retention volumes (VN) of isomer series on DBA (Surface adsorption region).

**Figure 2:**
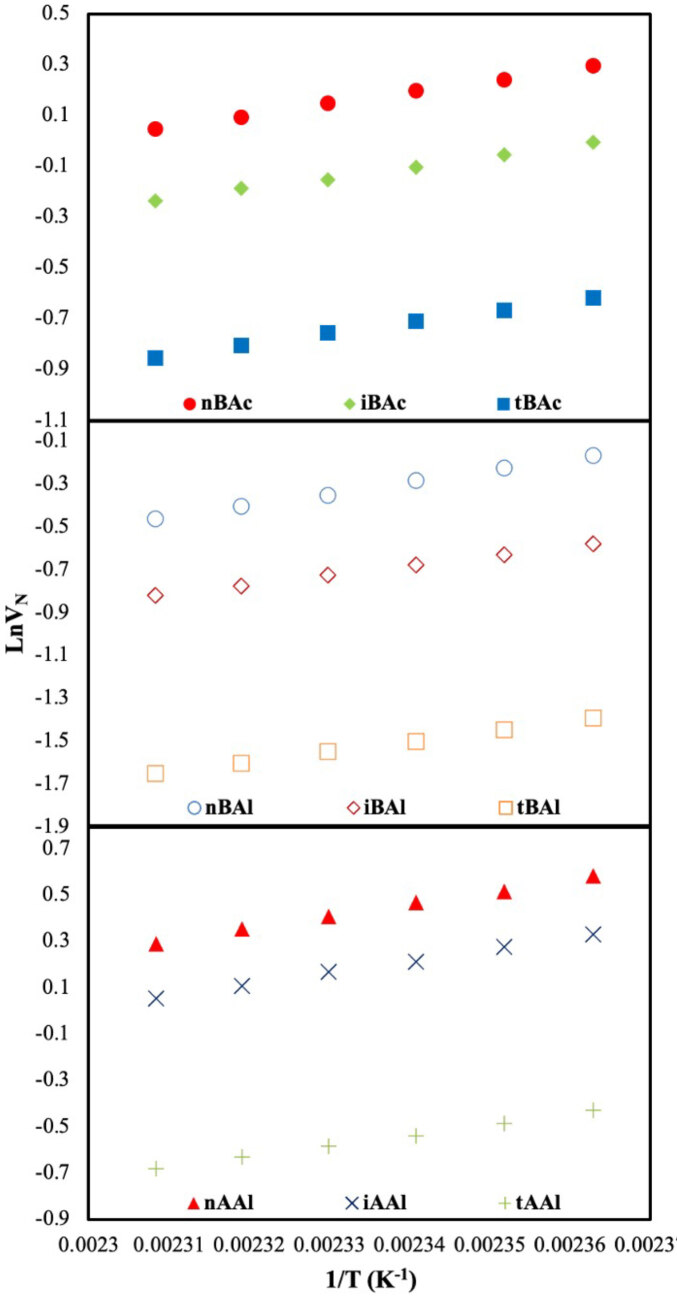
Net retention volumes (VN) of isomer series on DBA (Thermodynamic region).

The main data ( *V* *_N_* ) obtained from IGC-ID studies were calculated for all probes injected surface adsorption region (303.2–328.2 K) and thermodynamic region (423.2–433.2 K) according to Eq. (1). The retention diagrams of DBA used as separator stationary phase in two regions were given in Figure 1 and 2, respectively. The “a *”* values for nBAc/iBAc, nBAc/tBAc, nBAl/iBAl, nBAl/tBAl, nAAl/iAAl, and nAAl/tAAl were obtained using their *V* *_N_* in two regions. “a” values calculated according to Eq. (2) determined the separation ability of DBA. The higher the values of the separation factor calculated according to Eq. (2), the better the selectivity for isomers. Table 3 and 4 shows the calculated the values for the isomer pairs in two regions. Considering these values, it is seen that isomers are separated. Besides, it was observed that structural isomers were better separated in the surface adsorption region than in the thermodynamic region. 

**Table 3:** The separation factor of DBA for the isomer pairs: nBAc/iBAc, nBAc/tBAc, nBAl/iBAl, nBAl/tBAl, nAAl/iAAl, and nAAl/tAAl (303.2–328.2 K).

a = VN1 / VN2						
T (K)	VNnBAc / VNiBAc	VNnBAc / VNtBAc	VNnBAl / VNiBAl	VNnBAl / VNtBAl	VNnAAl / VNiAAl	VNnAAl / VNtAAl
303.2	1.52	3.99	1.72	6.26	1.34	6.49
308.2	1.55	4.15	1.58	6.69	1.31	6.19
313.2	1.57	3.98	1.60	6.66	1.48	6.20
318.2	1.57	4.00	1.65	7.41	1.35	6.03
323.2	1.58	3.90	1.70	7.89	1.46	5.97
328.2	1.57	3.88	1.74	8.34	1.35	5.46

**Table 4:** The separation factor of DBA for the isomer pairs: nBAc/iBAc, nBAc/tBAc, nBAl/iBAl, nBAl/tBAl, nAAl/iAAl, and nAAl/tAAl (423.2–433.2 K).

a = VN1 / VN2						
T (K)	VNnBAc / VNiBAc	VNnBAc / VNtBAc	VNnBAl / VNiBAl	VNnBAl / VNtBAl	VNnAAl / VNiAAl	VNnAAl / VNtAAl
423.2	1.35	2.50	1.52	3.40	1.29	2.75
425.2	1.35	2.51	1.51	3.33	1.27	2.71
427.2	1.36	2.49	1.49	3.45	1.29	2.76
429.2	1.35	2.47	1.45	3.38	1.28	2.70
431.2	1.33	2.44	1.46	3.35	1.28	2.70
433.2	1.33	2.44	1.43	3.32	1.26	2.63

**Figure 3:**
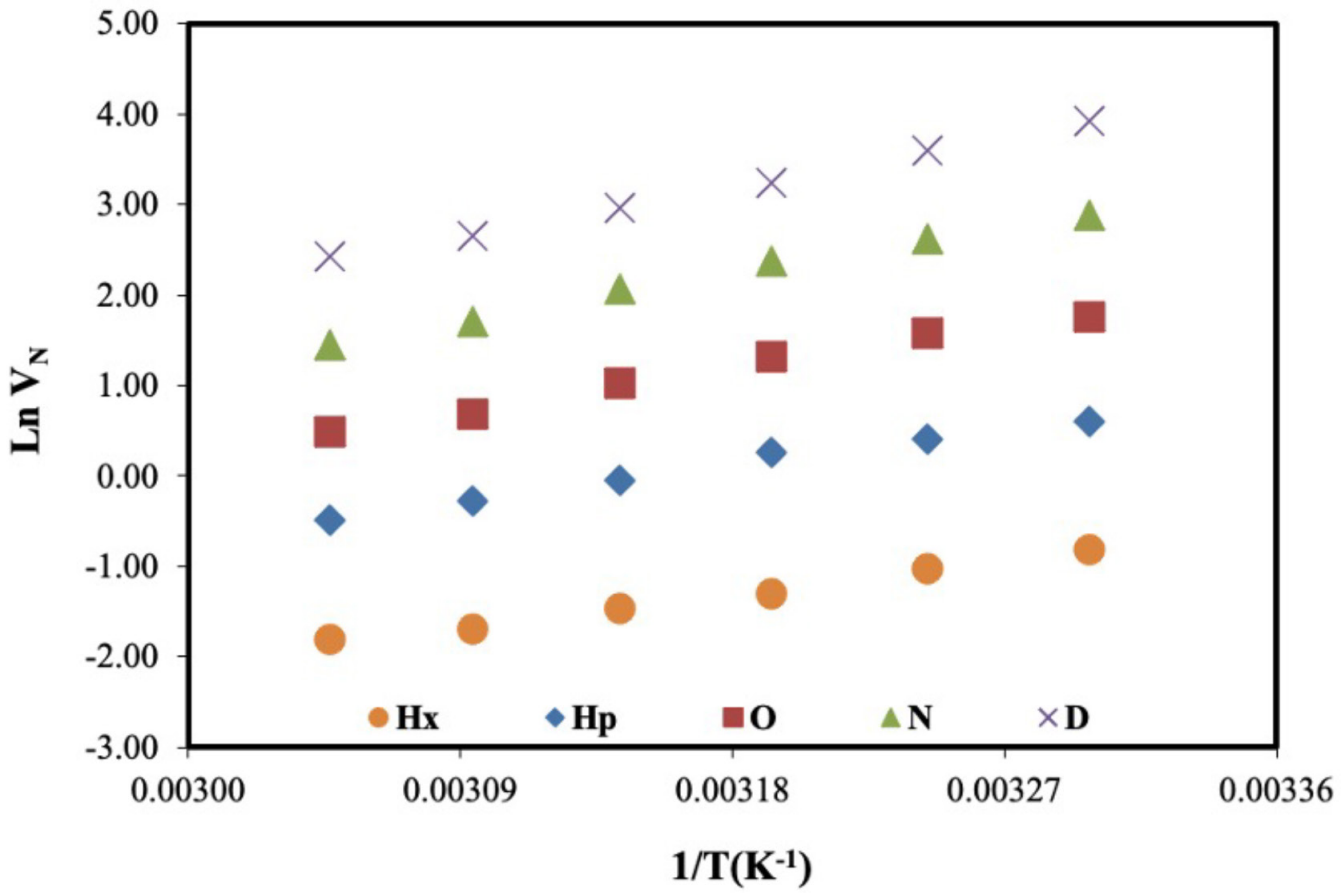
Net retention volumes (VN) of nonpolar probes on DBA.

**Figure 4:**
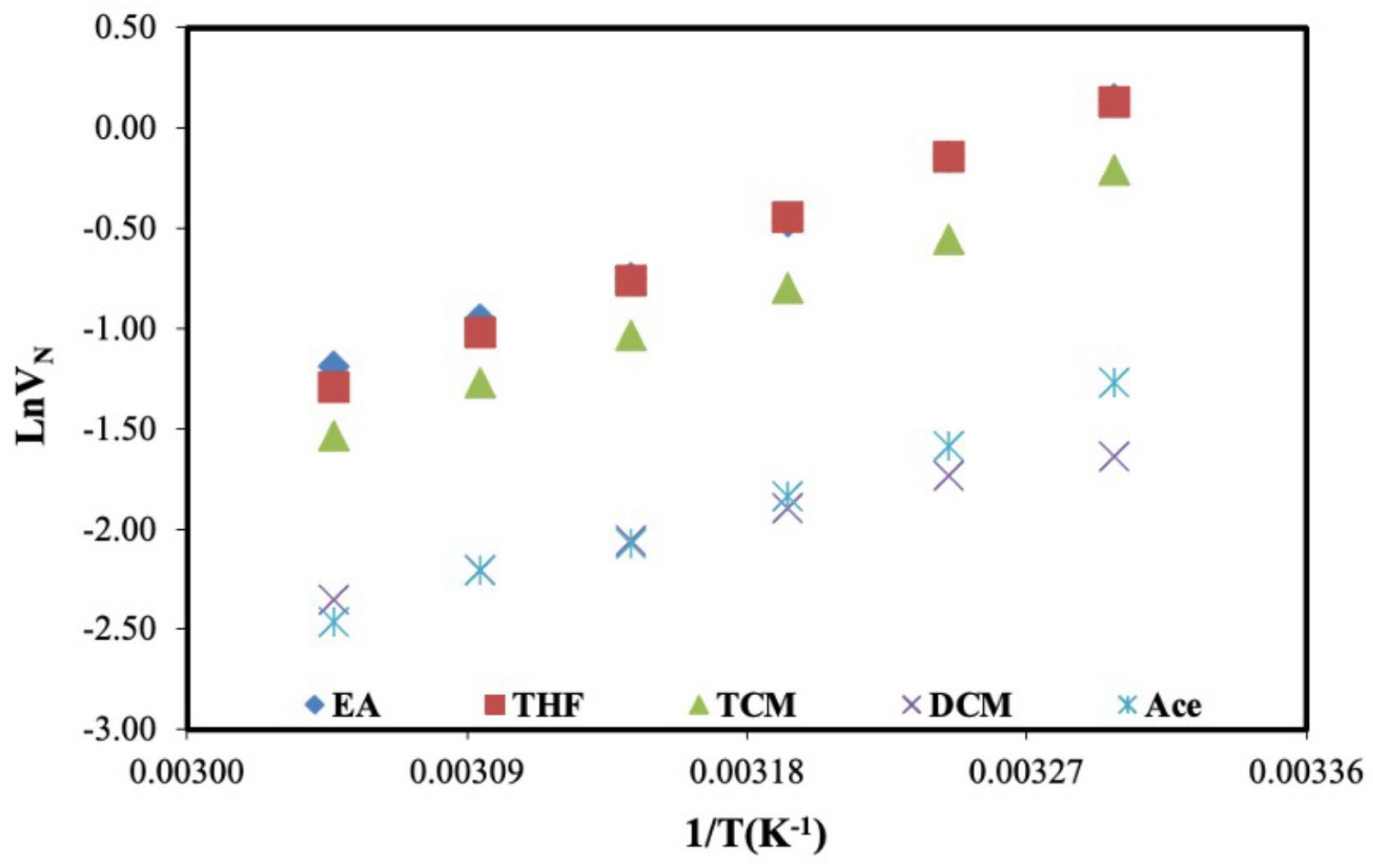
Net retention volumes (VN) of polar probes on DBA.

The main data ( *V* *_N_* ) obtained from IGC-ID studies was calculated for all probes between 303.2 and 328.2 K according to Eq. (1). Retention diagrams of non-polar and polar probes were given in Figure 3 and 4, respectively.

**Figure 5:**
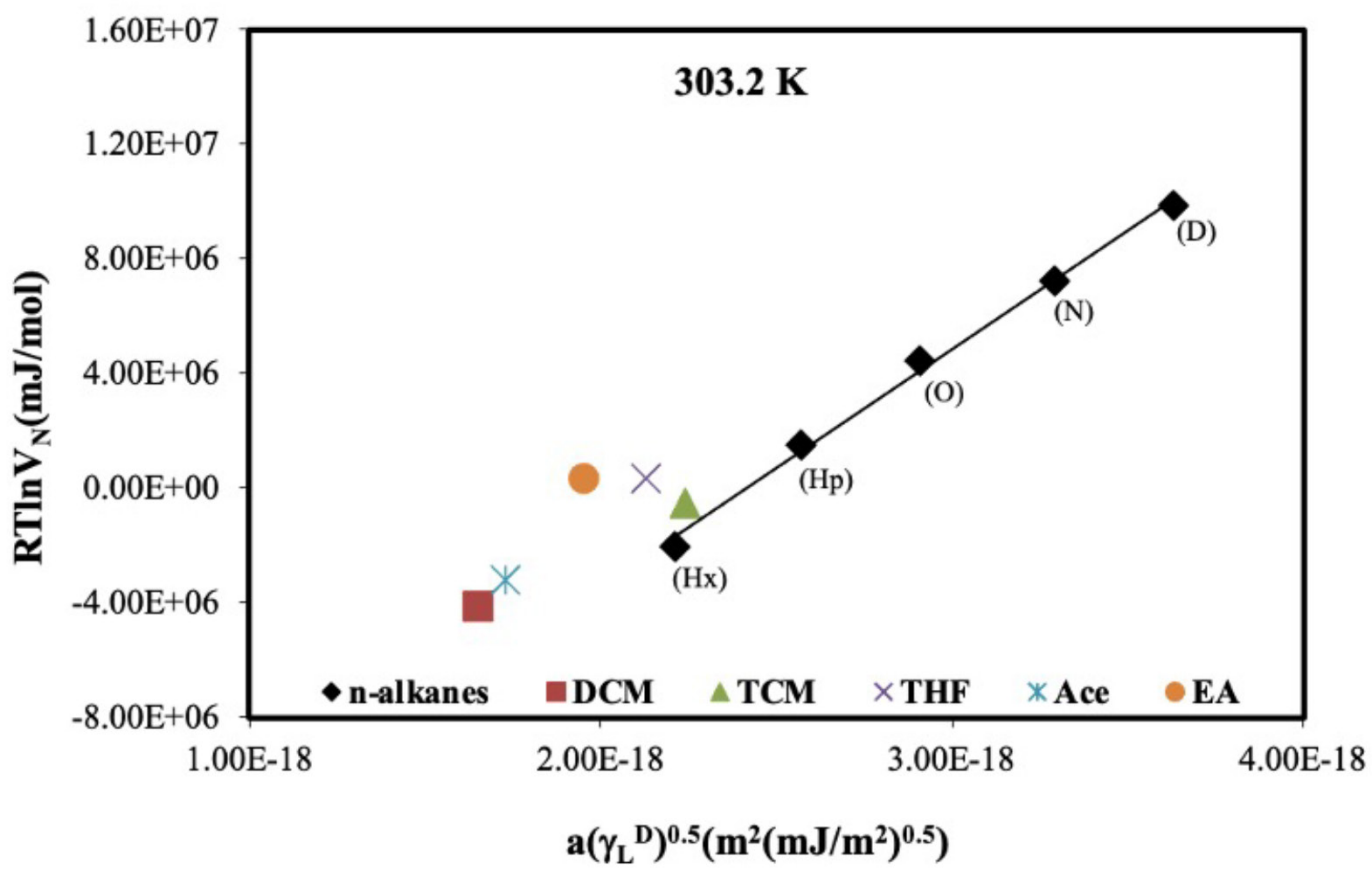
A linear plot of RTlnVN vs a(gL D)0.5 for all probes on DBA at 303.2 K.

The surface energy of a solid materials depends on the chemical structure, physical properties, and composition. Interactions between molecules on a solid surface and polar or nonpolar probe molecules are due to long- and short-range interactions known as weak interactions (London dispersive forces) and strong interactions (acid-base interactions). Dispersive surface energy occurs as a result of nonspecific interactions caused by the London dispersive forces known as weak or long-range interactions [54]. g_S_ *^D^* can be calculated using IGC-ID technique based on well-known approaches for data analysis, such as Dorris–Gray (Eq. (5)) and Schultz (Eq. (7)) methods. In these a_S_ *^D^* calculations, homologous alkane vapor series are used in infinite dilution, resulting in a single numerical a_S_ *^D^* value. D *G* *_A_* for the all probes were calculated from the Schultz method using Eq. (7) in the surface adsorption region (303.2–328.2 K). A plot of *RTlnV* *_N_* versus *a(* g_L_ *^D^* *)* *^0.5^* for all probes was plotted at 303.2 K in Figure 5. From Eqs. (5) and (7), g_S_ *^D^* of DBA was calculated using Schultz and Dorris-Gray methods. The results obtained from studies were listed in Table 5.

**Table 5:** Dispersive surface energy (gSD) of DBA.

	gSD mj.m–2	
T (K)	Schultz	Dorris-Gray
303.2	47.51	47.74
308.2	47.06	47.69
313.2	46.38	47.36
318.2	45.89	47.27
323.2	44.71	46.45
328.2	44.06	46.19

It is showed that the value determined for g_S_ *^D^* of DBA have different ranges from 47.51–44.06 (Schultz method) to 47.74–46.19 mj/m^2^ (Dorris-Gray method). Besides, it is observed that the g_S_ *^D^* values calculated by Dorris-Gray method are higher than those obtained from the Schultz method. The g_S_ *^D^* values obtained from the Schultz method decrease faster than the g_S_ *^D^* values obtained by the Dorris–Gray method with increasing temperature. The results obtained for the Schultz and Dorris–Gray method at surface adsorption region are close to each other, showing that these two methods are compatible and feasible. There is no study on DBA in the literature. A rough comparison can be made with reported LCs. In the literature, g_S_ *^D^* values for LCs were ranged from 30 to 42 mj/m^2^ in agreement with this study [50, 55]. 

The values of *–* D *G* *_A_* *^S^* were calculated by the numerical difference between the calculated value of *RTlnV* *_N_* and that which was obtained from Eq. (7) of the linear plot of the nonpolar reference line. The variation of D *G* *_A_* *^S^* between DBA and the polar probes for the studied temperatures is given in Table 6. Regarding the Table 6, it was seen that the temperature did not change the D *G* *_A_* *^S^* values much. D *H* *_A_* *^S^* values were calculated for polar probes and the results were given in Table 7. The D *H* *_A_* *^S^* values were calculated as the degree of interaction between the DBA molecule surface and the polar probe molecules. These values were followed the order THF>EA>Ace>TCM>DCM. The DCM probe molecule ( *DN* = 0.0, *AN* = 16.4) showed the lowest *–* D *H* *_A_* *^S^* value which is to be expected taking into account the acidic properties of this molecule and the acidic properties of LC surface given by the *K* *_A_* value. THF is a basic probe molecule ( *DN* = 84.4, *AN* = 2.1), it may be expected to interact strongly with acid surfaces [56,57]. Considering the values of D *H* *_A_* *^S^* and D *G* *_A_* *^S^* for each polar probe, adsorption occurs exothermically and spontaneously for all studied temperatures. The specific intermolecular interactions are derived from the interaction between the polar probe and the Lewis acidic-basic sites on surface [58–60]. 

**Table 6:** The variation of free energy of specific interactions, –DGAS (kj/mol), between DBA and the polar probes for the studied temperatures.

T (K)	EA	Ace	DCM	TCM	THF
303.2	4.21	2.48	2.21	0.91	2.69
308.2	3.99	2.10	2.39	0.54	2.50
313.2	3.68	1.92	2.42	0.45	2.28
318.2	3.54	1.83	2.53	0.42	2.03
323.2	3.63	2.03	2.67	0.44	2.00
328.2	2.95	1.24	2.17	-0.34	1.21

**Table 7:** Values of enthalpy (DHAS) of adsorption on DBA for the polar probes.

Polar probes	–DGAS (kj/mol)
Ace	13.68
EA	17.20
THF	18.39
DCM	0.87
TCM	12.12

**Figure 6:**
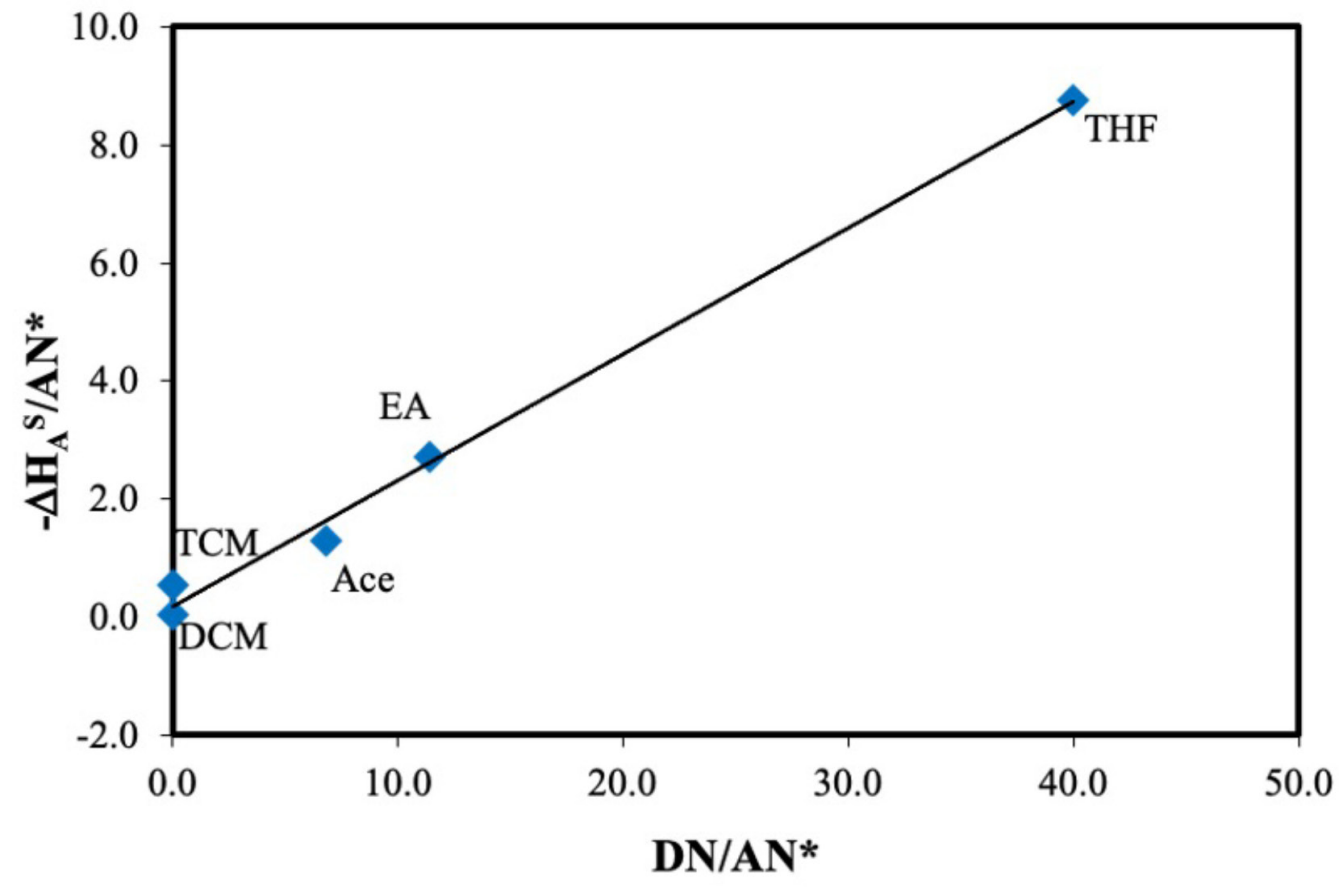
The plot of –DHA S/AN* vs DN/AN*.

A plot of *–* D *H* *_A_* *^S^* /AN* versus DN/AN* was plotted by *K* *_A_* as the slope and *K* *_D_* as the intercept using Eq. (11), and it is shown in Figure 6. The character of DBA surface was determined by the ratio of *K* *_D_* */K* *_A_* . The obtained *K* *_A_* and *K* *_D_* values were listed in Table 8. Due to the *K* *_D_* */K* *_A_* value is lower than 1, DBA surface is an acidic character. 

**Table 8:** Lewis acid-base parameters, KA and KD, of DBA.

Liquid crystal	KA	KD	KD/ KA
DBA	0.2134 ± 0.012	0.1907 ± 0.009	0.89

## 5. Conclusion

IGC-ID technique was used to investigate the separation of isomer series in surface adsorption (303.2–328.2 K) and thermodynamic region (423.2–433.2 K) and the surface properties of DBA in surface adsorption region. Considering the separation factors, it was determined that the DBA in IGC-ID technique can be used to separate the isomer series in surface adsorption and thermodynamic region. The values of g_S_ *^D^* for DBA were determined to be 47.51–44.06 mj/m^2^ using the Schultz method and 47.74–46.19 mj/m^2^ using the Dorris–Gray method. g_S_ *^D^* values from both calculation methods decrease linearly with the increase in temperature in the range from 303.2 to 328.2 K. The values of *K* *_A_* and *K* *_D_* were found to be 0.2134 and 0.1907, respectively. As shown that, the *K* *_D_* value is lower than the *K* *_A_* . In this case, it can be said that the DBA surface is an acidic character. The IGC-ID technique is very important in improving the quality of products for industrial fields, since isomers can be separated effectively and the surface energy of samples can be easily determined. 
